# A Multi-Hospital Comparative Study on the Efficacy of Probiotics Versus Placebo in Preventing Antibiotic-Associated Diarrhea in Adult Patients

**DOI:** 10.7759/cureus.70881

**Published:** 2024-10-05

**Authors:** Jamal Shah, Samran Hasan Adnan, Mian Shah Yousaf, Syed Jamal Ud Din, Moeen Ul Haq, Nadia Siddiqui, Minahil Shahid, Ahmed Samir Abdul Elhamid, Shiza Rauf, Muhammad Afnan

**Affiliations:** 1 General Internal Medicine, Khyber Teaching Hospital, Peshawar, PAK; 2 Medicine, Dow International Medical College, Karachi, PAK; 3 Gastroenterology, Prime Teaching Hospital, Peshawar, PAK; 4 Respiratory Medicine, Queen Elizabeth University Hospital, Glasgow, GBR; 5 Gastroenterology, Mufti Mahmood Memorial Teaching Hospital and Gomal Medical College, Dera Ismail Khan, PAK; 6 Internal Medicine, Jinnah Medical and Dental College, Karachi, PAK; 7 Medicine/Surgery, Shalamar Medical and Dental College, Lahore, PAK; 8 Gastroenterology and Hepatology, Faculty of Medicine, Delta University for Science and Technology, Mansoura, EGY; 9 Medicine, Leicester Royal Infirmary, Leicester, GBR; 10 Cardiovascular Medicine, Khyber Institute of Medical Sciences, Kohat, PAK

**Keywords:** antibiotic-associated diarrhea, bifidobacterium, gastrointestinal health, lactobacillus, placebo, probiotics

## Abstract

Background: Antibiotic-associated diarrhea (AAD) is a prevalent complication of antibiotic therapy, attributed to disruptions in gut microbiota. Probiotics are increasingly studied for their potential in preventing AAD by restoring microbial balance.

Objective: The aim of this investigation was to assess the efficacy of probiotics in reducing AAD in adult patients when compared to a placebo.

Methodology: This research was conducted from March 2023 to March 2024 using a randomized, placebo-controlled design at multiple institutions: Khyber Teaching Hospital, Peshawar; Prime Teaching Hospital, Peshawar; Mufti Mahmood Memorial Teaching Hospital, Dera Ismail Khan; Shalamar Hospital, Lahore; University Hospitals of Leicester; and DHQ Teaching Hospital Kohat, enrolling 340 adult patients prescribed systemic antibiotics. Eligible participants were aged 18 years and older, while those with chronic diarrhea, inflammatory bowel disease, immunodeficiency, recent probiotic or antibiotic use, or inability to provide informed consent were excluded. The sample size was calculated using WHO guidelines, resulting in a target of 340 to ensure adequate power. Participants were randomized to receive either probiotics (Lactobacillus rhamnosus GG and Bifidobacterium longum BB536) or placebo, administered within 24 hours of starting antibiotics. Daily monitoring of bowel habits and symptoms was performed using standardized diaries, and adherence was evaluated through pill counts. Statistical analyses were performed using IBM SPSS Statistics for Windows, Version 27 (Released 2020; IBM Corp., Armonk, New York, United States), comparing the incidence, severity, and duration of AAD between groups, with a significance threshold of p < 0.05.

Results: In the probiotic group, 31 patients (18.23%) developed AAD compared to 53 patients (31.17%) in the placebo group (p=0.01). Among those with AAD, the probiotic group experienced a shorter duration (mean 3.5 ± 1.2 days) compared to placebo (mean 5.1 ± 1.8 days, p=0.002). Adherence rates were high in both groups (probiotic: 96.4%, placebo: 95.9%). Significant improvements in bowel habits were reported more frequently in the probiotic group (77.06%) than placebo (50.59%, p=0.02). The hospital stay duration was similar between groups (probiotic: mean 7.8 ± 2.1 days, placebo: mean 8.3 ± 2.4 days, p=0.15).

Conclusion: Probiotics significantly reduced the incidence and duration of AAD compared to placebo, with high adherence and favorable patient-reported outcomes.

## Introduction

A large percentage of patients (35%) receiving antibiotic therapy suffer from antibiotic-associated diarrhea (AAD), which presents a challenging therapeutic scenario [1,2]. The primary source of the disturbance produced by antibiotics is the imbalance in the gut microbiota that characterizes this illness [3]. Because probiotics can balance the gut microbiota and lessen diarrhea symptoms, their use as a preventive strategy against AAD has gained traction [4]. However, even after a plethora of research examining this strategy, opinions on whether probiotics are more effective than a placebo at preventing AAD in adult patients are still divided [5,6].

Antibiotics attack both good and bad bacteria in the stomach without distinction, even though they are necessary for treating bacterial infections [7]. Gastrointestinal symptoms are frequently brought on by this disturbance, with diarrhea being the most typical symptom [8]. Probiotics represent a potentially effective path for intervention, as they are described as live microorganisms that, when given in sufficient proportions, bestow health advantages to the host [9]. These microbes, which are mostly Lactobacillus and Bifidobacterium strains, are thought to restore the gut flora and maybe lessen the frequency and severity of AAD [10].

There have been mixed findings from earlier studies on the effectiveness of probiotics in preventing AAD [11,12]. Probiotics have been shown in several studies to significantly lower the incidence of diarrhea, indicating their potential benefit in preserving gut health while undergoing antibiotic medication [13,14]. On the other hand, other research has not consistently shown benefits, raising questions about the best probiotic strains, dosage, and patient demographics [15,16].

Resolving these disparities is essential to improving patient outcomes and clinical practice. In order to provide solid information to support clinical decision-making, this research will compare probiotics with placebo in adult patients receiving antibiotic therapy. Probiotics' ability to prevent AAD will be methodically assessed in this study, which will use a randomized controlled trial design to reduce bias and guarantee accurate results.

Research objective

The aim of this investigation was to assess the efficacy of probiotics in reducing AAD in adult patients when compared to a placebo.

## Materials and methods

Study design and settings

This research was conducted from March 2023 to March 2024 using a randomized, placebo-controlled design at multiple institutions: Khyber Teaching Hospital, Peshawar; Prime Teaching Hospital, Peshawar; Mufti Mahmood Memorial Teaching Hospital, Dera Ismail Khan; Shalamar Hospital, Lahore; University Hospitals of Leicester; and DHQ Teaching Hospital Kohat.

Inclusion and exclusion criteria

Participants eligible for inclusion were adult patients (aged 18 years and above) and those prescribed systemic antibiotics for therapeutic purposes. Exclusion criteria included patients with a history of chronic diarrhea, inflammatory bowel disease, immunodeficiency disorders, recent probiotic or antibiotic use (within 30 days), and those unable to provide informed consent.

Sample size

The sample size for the study was calculated using the following formula from the World Health Organization (WHO) [17]:

n = z^2^ x p x (1 - p)\e^2^

where n: required sample size; z: Z-value for the desired confidence level (1.96 for a 95% confidence level); p: estimated proportion of the population with the outcome (e.g., incidence rate of AAD); e: margin of error (desired precision, typically set at 0.05).

We estimated p to be 0.3 (30%) and e was set at 0.05.

n =1.962 x 0.3 x (1 - 0.3)\0.052

n =3.8416 x 0.21\0.0025

n = 0.806496\0.0025

n ≈322.6

The calculated sample size was approximately 322.6. To ensure adequate power and account for potential dropouts, the study included a total of 340 participants.

Study protocol and probiotic administration

Upon admission, demographic data (age and gender) and baseline clinical characteristics (including underlying conditions requiring antibiotic therapy and the type of antibiotics prescribed) were recorded. Participants were randomly assigned to receive either probiotics (Lactobacillus rhamnosus GG and Bifidobacterium longum BB536) or a placebo, using a computer-generated sequence to ensure equal distribution across both groups. Stratified randomization was applied based on age and underlying condition to maintain balance across key demographics. The study was double-blind, ensuring that both participants and healthcare providers were unaware of group assignments, with data analysts also blinded to minimize bias.

Probiotics were administered within 24 hours of starting antibiotic therapy and continued for the entire duration of the antibiotic regimen. Daily monitoring of bowel habits and symptoms was conducted using standardized diaries. Adherence to the treatment was evaluated through pill counts and participant self-reporting.

The probiotic formulation consisted of 1 × 10^10^ CFU per capsule, with participants in the probiotic group receiving one capsule twice daily, resulting in a daily dose of 2 × 10^10^ CFU. Standardization of administration was achieved by ensuring probiotics were given in parallel with the antibiotic regimen, with adherence monitored closely throughout the study to maintain consistency across participants.

Probiotic strain selection

The probiotic formulation used in this study consisted of Lactobacillus rhamnosus GG (LGG) and Bifidobacterium longum BB536. These strains were selected based on their well-documented efficacy in managing AAD and their recognized safety profile. Lactobacillus rhamnosus GG has been extensively studied for its ability to maintain gut barrier function and modulate immune responses, reducing the incidence of AAD. Bifidobacterium longum BB536 is known for its role in promoting gut health and restoring microbial balance disrupted by antibiotic treatment. Both strains are classified as Generally Recognized as Safe (GRAS) by the FDA and were used within recommended dosages.

Statistical analysis

Statistical analyses were conducted using IBM SPSS Statistics for Windows, Version 27 (Released 2020; IBM Corp., Armonk, New York, United States). Descriptive statistics were used to summarize baseline characteristics, including age, gender, underlying conditions, and types of antibiotics administered. Inferential statistics were applied to compare the incidence, severity, and duration of AAD between the two groups. Logistic regression was employed to calculate odds ratios (OR) with 95% confidence intervals (CI) for the incidence, severity, and duration of AAD. Statistical significance was assessed with p-values, with a threshold of p < 0.05 considered significant. Analyses also included comparisons of hospital stay duration, adherence to intervention, and reported side effects between the groups, using appropriate statistical tests such as the chi-square test for categorical variables and t-tests for continuous variables.

## Results

The study included 340 participants, evenly divided into the probiotic group (n=170) and the placebo group (n=170). Age distribution in the probiotic group was as follows: 18-30 years (25 patients, 14.71%), 31-45 years (50 patients, 29.41%), 46-60 years (60 patients, 35.29%), and 61+ years (35 patients, 20.59%), with a mean age of 45.2 ± 15.4 years. The placebo group had the following age distribution: 18-30 years (30 patients, 17.65%), 31-45 years (45 patients, 26.47%), 46-60 years (55 patients, 32.35%), and 61+ years (40 patients, 23.53%), with a mean age of 46.1 ± 14.8 years. Gender distribution was 87 males (51.18%) and 83 females (48.82%) in the probiotic group, and 81 males (47.65%) and 89 females (52.35%) in the placebo group. Regarding underlying conditions, the probiotic group had 50 patients (29.41%) with respiratory infections, 45 patients (26.47%) with urinary tract infections, 35 patients (20.59%) with skin infections, and 40 patients (23.53%) with other conditions. The placebo group had 55 patients (32.35%) with respiratory infections, 40 patients (23.53%) with urinary tract infections, 30 patients (17.65%) with skin infections, and 45 patients (26.47%) with other conditions. The types of antibiotics administered were penicillin (50 patients, 29.41%), cephalosporin (60 patients, 35.29%), macrolides (25 patients, 14.71%), and fluoroquinolones (35 patients, 20.59%) in the probiotic group, and penicillin (55 patients, 32.35%), cephalosporin (65 patients, 38.24%), macrolides (30 patients, 17.65%), and fluoroquinolones (20 patients, 11.76%) in the placebo group (Table 1).

**Table 1:** Baseline demographic and clinical characteristics.

Characteristic		Probiotic Group (n=170)	Placebo Group (n=170)
Age Groups (n;%)	18-30 years	25 (14.71)	30 (17.65)
	31-45 years	50 (29.41)	45 (26.47)
	46-60 years	60 (35.29)	55 (32.35)
	61+ years	35 (20.59)	40 (23.53)
	(mean ± SD)	45.2 ± 15.4	46.1 ± 14.8
Gender (n;%)	Male	87 (51.18)	81 (47.65)
	Female	83 (48.82)	89 (52.35)
Underlying Conditions (n;%)	Respiratory Infection	50 (29.41)	55 (32.35)
	Urinary Tract Infection	45 (26.47)	40 (23.53)
	Skin Infection	35 (20.59)	30 (17.65)
	Others	40 (23.53)	45 (26.47)
Type of Antibiotics (n;%)	Penicillin	50 (29.41)	55 (32.35)
	Cephalosporin	60 (35.29)	65 (38.24)
	Macrolides	25 (14.71)	30 (17.65)
	Fluoroquinolones	35 (20.59)	20 (11.76)

**Figure 1:**
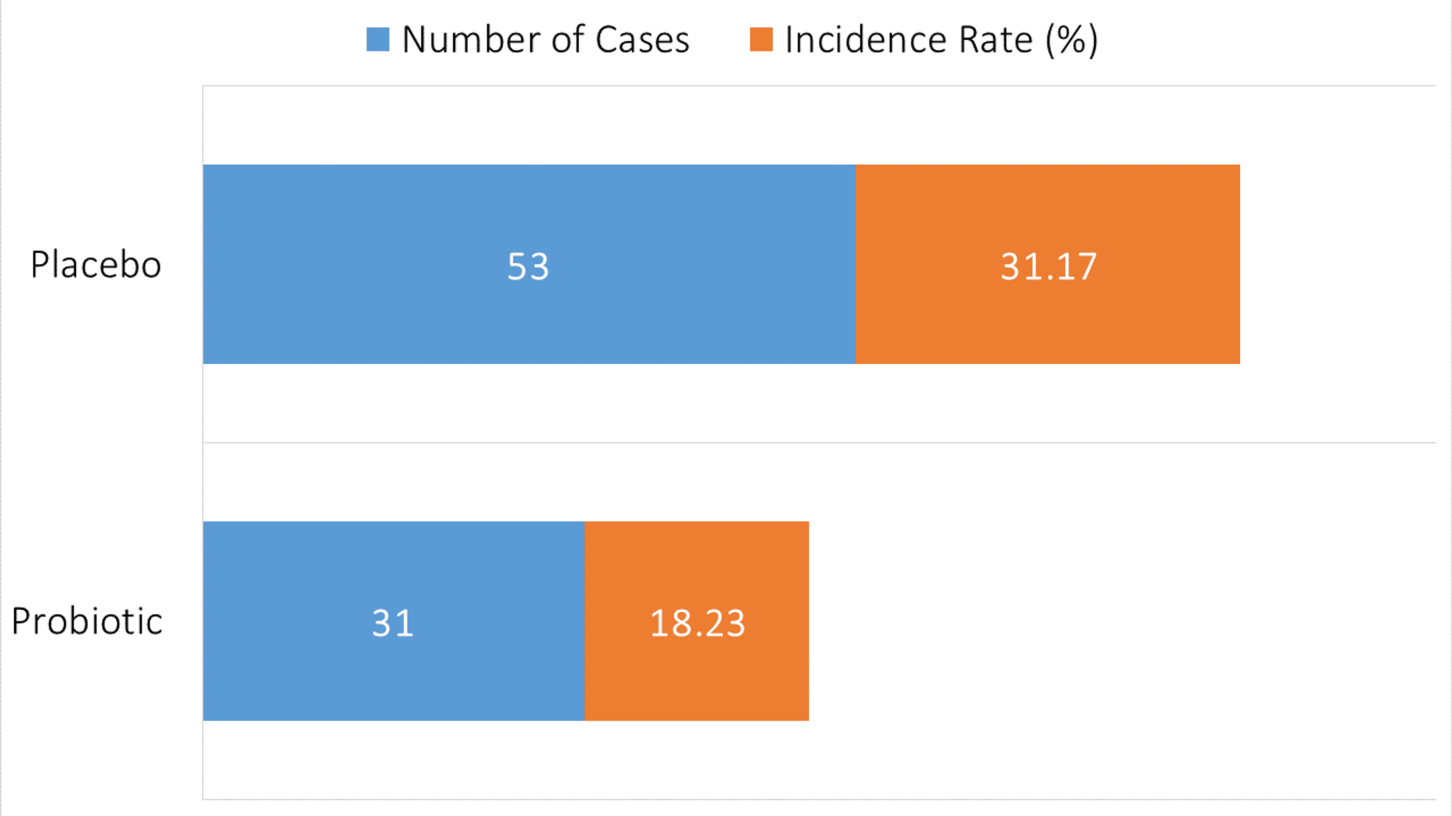
Incidence of antibiotic-associated diarrhea.

In the probiotic group, 31 out of 170 patients (18.23%) developed AAD. In contrast, the placebo group had 53 out of 170 patients (31.17%) who experienced AAD (Figure 1).

**Figure 2:**
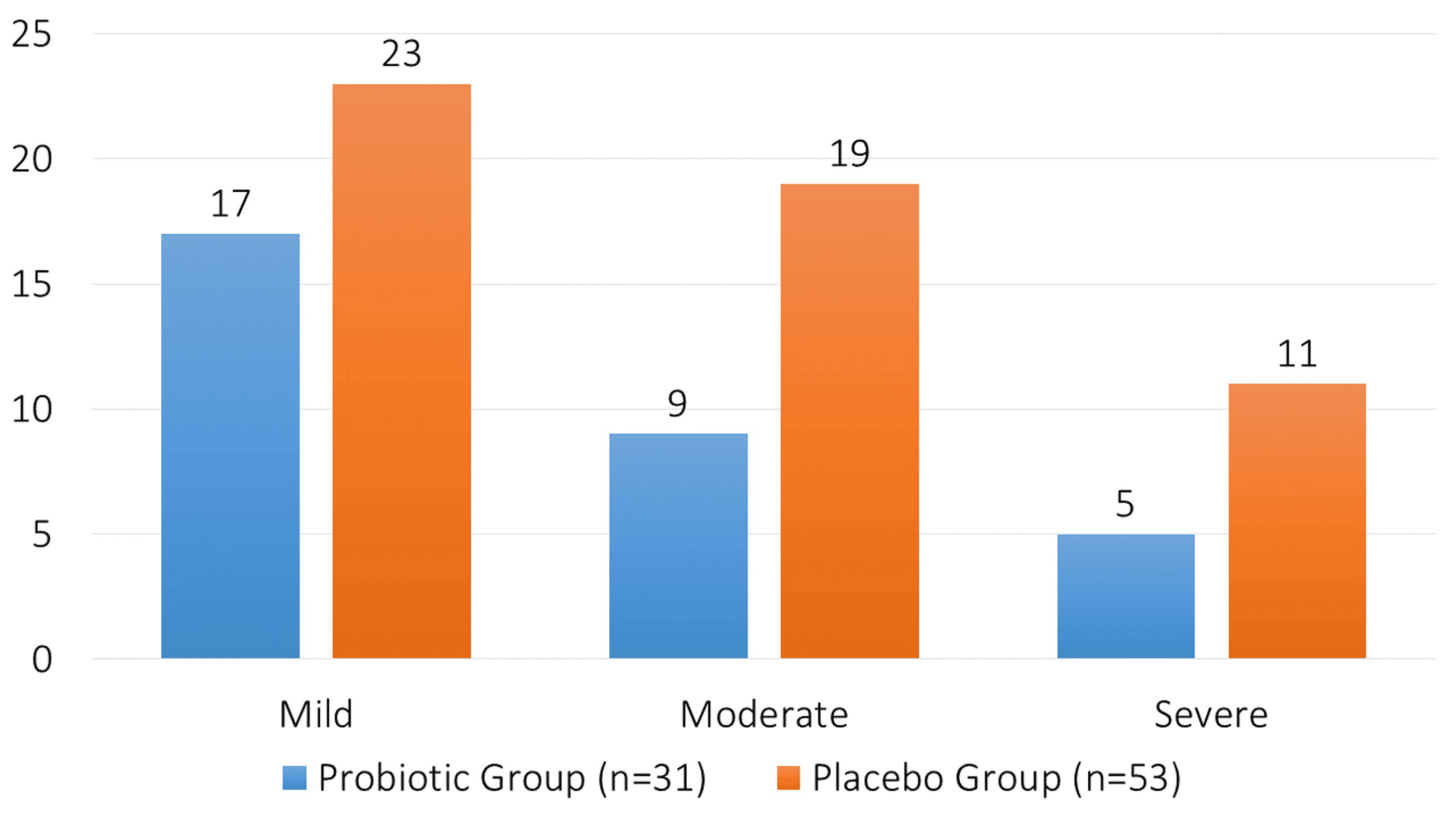
Severity of diarrhea.

Among the 31 patients in the probiotic group who developed AAD, 17 cases (54.84%) were mild, nine cases (29.03%) were moderate, and five cases (16.13%) were severe. In the placebo group, of the 53 patients who developed diarrhea, 23 cases (43.40%) were mild, 19 cases (35.85%) were moderate, and 11 cases (20.75%) were severe (Figure 2).

The probiotic group had an average AAD duration of 3.5 ± 1.2 days, with a median of 3 days (interquartile range (IQR): 2-4 days). The mean duration in the placebo group was 5.1 ± 1.8 days, and the median was 5 days (IQR: 3-6 days). Between the two groups, there was a statistically significant difference in the length of diarrhea (p=0.002) (Table 2).

**Table 2:** Duration of diarrhea and adherence to intervention.

Group	Mean Duration ± SD (days)	Median Duration (IQR)	p-value	Mean Pill Count ± SD	Adherence Rate (%)
Probiotic	3.5 ± 1.2	3 (2-4)	0.002	95.5 ± 4.2	96.4
Placebo	5.1 ± 1.8	5 (3-6)		94.8 ± 5.1	95.9

In the probiotic group, 131 patients (77.06%) reported an improvement in bowel habits, 26 patients (15.29%) reported no change, and 13 patients (7.65%) reported worsened bowel habits (Table 3). In the placebo group, 86 patients (50.59%) reported an improvement, 46 patients (27.06%) reported no change, and 38 patients (22.35%) reported worsened bowel habits. The difference between the groups was statistically significant (p=0.02).

**Table 3:** Patient self-reporting on bowel habits.

Group	Improvement Reported (n;%)	No Change (n;%)	Worsened (n;%)	p-value
Probiotic	131 (77.06)	26 (15.29)	13 (7.65)	0.02
Placebo	86 (50.59)	46 (27.06)	38 (22.35)	

**Figure 3:**
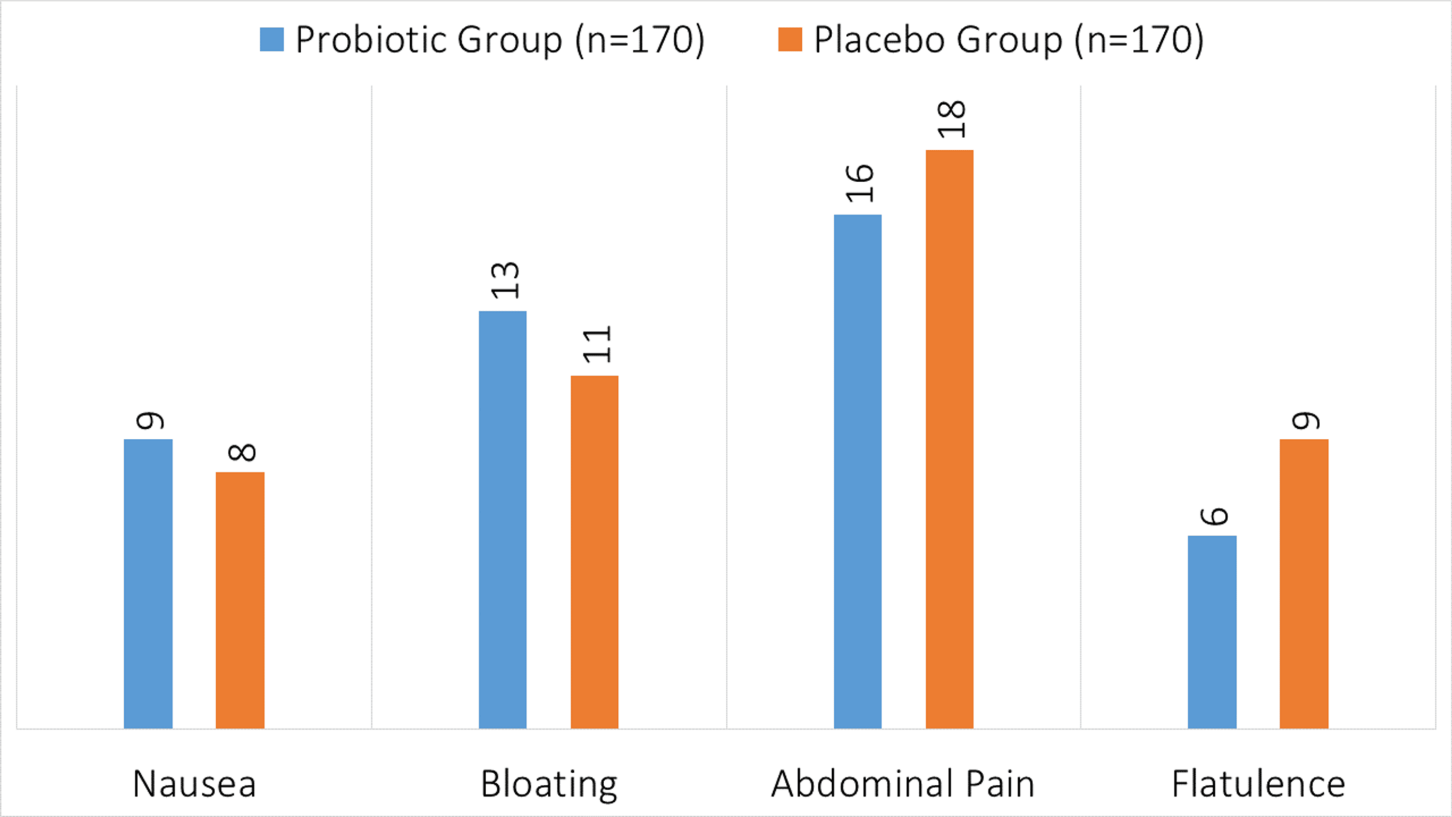
Side effects of probiotic and placebo.

In the probiotic group (n=170), the reported side effects were nausea in nine patients, bloating in 13 patients, abdominal pain in 16 patients, and flatulence in six patients (Figure 3). In the placebo group (n=170), the side effects included nausea in eight patients, bloating in 11 patients, abdominal pain in 18 patients, and flatulence in nine patients.

Table 4 shows that the probiotic group's hospital stay lasted an average of 7.8 ± 2.1 days, with a median stay of 8 days (interquartile range (IQR): 6-9 days). The mean duration for the placebo group was 8.3 ± 2.4 days, and the median was 8 days (IQR: 7-10 days). There was no statistically significant difference in the length of hospital stays between the two groups (p=0.15).

**Table 4:** Hospital stay duration.

Group	Mean ± SD (days)	Median (IQR)	p-value
Probiotic	7.8 ± 2.1	8 (6-9)	0.15
Placebo	8.3 ± 2.4	8 (7-10)	

The study findings indicate a statistically significant decrease in the incidence of diarrhea in the probiotic group compared to the placebo group (Table 5). The OR for the incidence of diarrhea in the probiotic group was 0.51 with a 95% confidence interval (CI) of 0.31 to 0.83 and a p-value of 0.01. There was no significant difference between the groups for the severity of diarrhea (moderate/severe vs. mild), as shown by the OR of 0.89, 95% CI of 0.54 to 1.48, and p-value of 0.65. With a p-value of 0.002, the OR for the length of diarrhea (≥3 days vs. <3 days) was 0.45, 95% CI of 0.27 to 0.74, and a significant decrease in the duration of diarrhea in the probiotic group.

**Table 5:** Inferential statistics for diarrhea incidence, severity, and duration.

Outcome	Odds Ratio (OR)	95% Confidence Interval (CI)	p-value
Incidence of Diarrhea	0.51	0.31 - 0.83	0.01
Severity of Diarrhea (Moderate/Severe vs. Mild)	0.89	0.54 - 1.48	0.65
Duration of Diarrhea (≥3 days vs. <3 days)	0.45	0.27 - 0.74	0.002

## Discussion

In this study, we explored the efficacy of probiotics in managing AAD among adult patients. It is important to highlight that probiotics have been utilized for decades, supported by previous studies demonstrating their benefits in gut health and prevention of AAD [2,3,5,9]. This long-standing history underpins our findings and underscores the relevance of our research in contributing to existing knowledge. Our results reinforce the established role of probiotics, specifically Lactobacillus rhamnosus GG and Bifidobacterium longum BB536, in promoting gut health during antibiotic therapy, providing further evidence for their clinical application. Due to the disruption of gut microbiota balance, AAD is a common side effect of antibiotic treatment, affecting approximately 35% of patients [1]. The purpose of this research was to test the effectiveness of probiotics against a placebo in preventing AAD in adult patients receiving antibiotic therapy. Our research offers important new information on the possible advantages of probiotics in therapeutic settings.

Compared to the placebo group (31.17%), the probiotic group had a significantly decreased incidence of AD (18.23%), with an OR of 0.51 (95% CI: 0.31-0.83, p=0.01). This decrease is consistent with other studies showing probiotics may lessen antibiotic-induced gut dysbiosis and the ensuing diarrhea [3]. Similar findings were found in a study by Hickson et al. who suggested that probiotic administration consistently reduced the prevalence of AAD in a variety of patient groups [18]. Our research did not identify a significant difference between the probiotic and placebo groups in moderate-to-severe instances of diarrhea (OR 0.89, 95% CI: 0.54-1.48, p = 0.65). This is in opposition to earlier research that suggested there may be advantages in lowering instances of severe diarrhea [19]. Interestingly, the probiotic group had diarrhea for 3.5 ± 1.2 days on average, whereas the placebo group experienced diarrhea for 5.1 ± 1.8 days on average (p=0.002). This research lends credence to the theory that probiotics hasten AAD recovery, maybe by improving immune response regulation and gut flora resilience. Similar outcomes have been seen in research supporting probiotics' ability to shorten the duration of diarrhea [20].

The probiotic group had a mean pill count of 95.5 ± 4.2 and an adherence rate of 96.4%, whereas the placebo group had a mean pill count of 94.8 ± 5.1 and an adherence rate of 95.9%. Both groups demonstrated excellent levels of adherence to the intervention. When interpreting results such as the considerable decrease in diarrhea occurrence and duration shown in the probiotic group, this high compliance implies excellent tolerance and acceptance of the probiotic regimen. Our results are corroborated by similar findings in another research study, which emphasizes the efficacy of probiotics in preventing AAD [21].

Probiotics have been shown to have a positive effect on bowel habits based on patient-reported outcomes. A substantial majority of participants in the probiotic group reported better symptoms than those in the placebo group (77.06% vs. 50.59%, p=0.02). This subjective evaluation supports the overall beneficial impact of probiotics on gastrointestinal health during antibiotic treatment, as it corresponds well with objective measurements of diarrhea occurrence and duration. These results are comparable with those of a prior research study [13], showing that probiotics may lower AAD and enhance patient-reported outcomes. Regarding safety, there was no significant difference in the frequency of mild side effects between the probiotic and placebo groups, including nausea, bloating, stomach discomfort, and flatulence. In particular, compared to eight patients who had nausea, 13 versus 11 experienced bloating, 16 versus 18 experienced gastrointestinal discomfort, and 6 versus 9 experienced flatulence. This result is consistent with previous research showing a largely positive safety profile for probiotics, with few cases of serious side effects [22].

Study limitations

While this research provides valuable insights into the effectiveness of probiotics in reducing AAD, several limitations should be considered. First, the study, though conducted across multiple hospitals in various regions, may still face limitations in generalizing the findings to broader, more diverse populations. Variations in regional microbiota and differing antibiotic use patterns across these locations could affect the generalizability of the specific probiotic strains and doses used. Second, despite employing participant self-reporting and pill counts to monitor adherence, variations in compliance across different sites might impact the overall results. Addressing these limitations in future research will be essential to enhance the efficacy and applicability of probiotic therapies in diverse clinical settings.

Importantly, this study lays the groundwork for future research by demonstrating that probiotics significantly reduce AAD incidence and duration. To further strengthen these findings, we recommend longer follow-up periods in future studies to assess the lasting effects of probiotics beyond the immediate post-antibiotic phase. This could provide valuable insights into any late-onset side effects or recurrences of AAD, ultimately enhancing the understanding and utility of probiotics in clinical practice.

## Conclusions

The effectiveness of probiotics in reducing AAD in adult patients is shown by this comparative investigation. When compared to a placebo, our results show a substantial decrease in the incidence and duration of AAD with probiotic treatment. Probiotics improved patient-reported outcomes and had a high adherence rate, showing their practical viability and acceptability in clinical settings, even though there was no discernible improvement in the severity of diarrhea. These findings support the regular use of probiotics during antibiotic treatment as a prophylactic intervention against AAD, possibly improving patient well-being and gastrointestinal health.

## Appendices

**Table 6:** Monitoring questionnaire for bowel habits and symptoms. The responses provided by participants were collected through standardized diaries, and the forms were filled out by researchers during follow-up assessments throughout the antibiotic treatment period.

Category	Questions	Response Type
Daily Bowel Habits	How many times did you have a bowel movement today?	Numeric
	Was your stool consistency normal, hard, loose, or watery?	Multiple Choice (Normal/Hard/Loose/Watery)
	Did you experience any urgency or discomfort when having a bowel movement today?	Yes/No
	Did you notice any blood or mucus in your stool today?	Yes/No
Bowel-Related Symptoms	Did you experience any abdominal pain or cramping today?	Yes/No
	How severe was the abdominal pain or cramping (if present)?	Numeric Scale (1-10)
	Did you feel bloated or have excessive gas today?	Yes/No
	Did you experience any nausea or vomiting today?	Yes/No
	Did you notice any changes in appetite today?	Yes/No
	Did you experience any heartburn or acid reflux today?	Yes/No
Probiotic/Placebo Adherence	Did you take your assigned probiotic/placebo as prescribed today?	Yes/No
	How many pills did you take today (if less than prescribed)?	Numeric
	Did you experience any side effects after taking the probiotic/placebo today?	Yes/No
Antibiotic-Related Monitoring	Did you take your antibiotic dose(s) as prescribed today?	Yes/No
	Did you experience any side effects from the antibiotic today?	Yes/No
	How severe were the side effects from the antibiotic (if any)?	Numeric Scale (1-10)
General Well-Being	How would you rate your overall energy level today?	Numeric Scale (1-10)
	Did you feel tired or fatigued today?	Yes/No
	Did you experience any other symptoms or changes in health today not related to bowel movements?	Yes/No (With Description)
Hospital Stay & Treatment Duration	Did you stay in the hospital overnight today?	Yes/No
	How many days have you been in the hospital since starting antibiotic treatment?	Numeric
	Did you require any additional medical treatments for your symptoms today (e.g., IV fluids, pain relief)?	Yes/No (With Description)
